# Salivary oxidative stress persists in inflammatory bowel disease regardless of biological treatment response

**DOI:** 10.3389/fphar.2025.1699252

**Published:** 2025-11-07

**Authors:** Elisabetta Bigagli, Tommaso Innocenti, Gabriele Dragoni, Francesco Pindozzi, Andrea Galli, Maura Lodovici, Cristina Luceri

**Affiliations:** 1 Department of Neurosciences, Psychology, Drug Research and Child Health (NEUROFARBA), Section of Pharmacology and Toxicology, University of Florence, Florence, Italy; 2 Gastroenterology Unit, IBD Referral Centre, Careggi University Hospital, Florence, Italy; 3 Department of Experimental and Clinical Biomedical Sciences, University of Florence, Florence, Italy

**Keywords:** IBD, biological therapy, oxidative stress, infliximab, vedolizumab, biomarkers

## Abstract

**Background:**

Uncontrolled oxidative stress contributes to the pathogenesis of inflammatory bowel disease (IBD), yet its association with disease activity and response to biological therapy, has never been studied in saliva. We investigated whether salivary oxidative stress markers could predict therapeutic response to biologics in IBD patients.

**Methods:**

Seventy-three IBD patients (46 ulcerative colitis (UC), 27 Crohn’s disease (CD)) eligible for infliximab or vedolizumab and 56 healthy controls (HC) were enrolled. Salivary advanced oxidation protein products (AOPPs), advanced glycated end-products (AGEs), and ferric reducing antioxidant power (FRAS) were measured at baseline and at week 26. Clinical response was assessed at weeks 26 and 52, and endoscopic activity at baseline and week 52.

**Results:**

Baseline AOPPs and AGEs were higher in IBD than HC (p < 0.0001), but only AOPPs distinguished mild from moderate-severe endoscopic activity (AUC 0.72; p < 0.05). Clinical response at week 26 was 77.8% in CD and 69.6% in UC, yet AOPPs remained stable from baseline. Endoscopic remission at week 52 occurred in 40.7% of CD and 23.9% of UC patients. Neither baseline nor 26-week AOPPs or AGEs predicted endoscopic improvement or remission.

**Conclusion:**

Salivary AOPPs reflect baseline disease severity but do not predict response to biologics. Persistent AOPPs accumulation despite clinical control suggests a decoupling between clinical remission and oxidative homeostasis. Understanding the drivers and clinical relevance of persistent AOPPs is needed before considering potential therapeutic applications, such as antioxidant-based adjunctive strategies or interventions targeting AOPP-mediated damage, to improve remission rates in IBD patients receiving advanced treatments.

## 1 Introduction

Inflammatory bowel disease (IBD), encompassing Crohn’s disease (CD) and ulcerative colitis (UC), is a chronic, relapsing-remitting condition characterized by inflammation of the gastrointestinal tract and epithelial injury, causing lifelong morbidity (Burisch et al., 2013).

Both clinical investigations and experimental models indicate that uncontrolled oxidative stress due to reactive oxygen species (ROS) overproduction and impaired antioxidant defences, perpetrates inflammatory stimuli and contributes to gut mucosal damage (Bourgonje et al., 2020; Muro et al., 2024). Among protein markers of oxidative damage, advanced oxidation protein products (AOPPs) form during inflammation through myeloperoxidase-mediated chlorination of proteins (Capeillère-Blandin et al., 2004), while advanced glycation end products (AGEs), produced endogenously or acquired through diet or smoking, result from non-enzymatic reactions between sugars and biomolecules (Phuong-Nguyen et al., 2023). Increasing evidence indicates that both AOPPs and AGEs-mediated toxicity contribute to the pathogenesis and progression of IBD (Phuong-Nguyen et al., 2023; Jiang et al., 2024; Shi et al., 2023; Xie et al., 2014).

We previously demonstrated that AOPPs and AGEs are increased in the serum of patients with complicated CD requiring surgery compared to healthy volunteers and that AGEs negatively correlate with mucosal barrier integrity (Luceri et al., 2019; Bigagli et al., 2023). Plasma AOPPs also correlate with disease activity in CD patients (Krzystek-Korpacka et al., 2008). Of note, a recent meta-analysis found that plasma AOPPs are among the most promising markers to distinguish patients with active and inactive IBD from healthy individuals (Tratenšek et al., 2024).

Saliva is emerging as a less-invasive alternative to plasma for clinical diagnosis and monitoring (Song et al., 2023), but in IBD, research is limited and findings on salivary oxidative stress/antioxidant status markers are inconsistent (Jahanshahi et al., 2004; Rezaie et al., 2006; Szczeklik et al., 2018; Janšáková et al., 2021).

Biological therapies and small molecules have greatly improved IBD management and patient outcomes, but long-term treatment response remains a challenge for many patients (Raine and Danese, 2022; Behr et al., 2024). Since these therapies suppress a dysfunctional immune response, a successful treatment would be expected to reduce both inflammation and oxidative damage. However, Bourgonje et al. (2019) found that serum free thiols, markers of antioxidant defences, were still reduced in patients with CD in clinical remission compared to healthy controls. This finding indicates that subclinical disease activity is characterized by impaired antioxidant defenses and persistent oxidative stress. However, the study did not include a prospective analysis of oxidative damage markers or investigate their potential role in predicting treatment response.

To fill this gap, we investigated whether 6 months of infliximab or vedolizumab treatment was able to modify salivary oxidative damage and antioxidant status in IBD patients and whether this correlated with drug response.

## 2 Materials and methods

### 2.1 Study subjects and protocol

In this prospective study, adult patients with moderately-to-severely active CD and UC who were initiating biological therapy with an anti-TNFα agent (Infliximab, IFX) or, in the case of UC, with an anti-integrin agent (Vedolizumab, VDZ), were enrolled at the IBD Referral Centre of Careggi University Hospital. This study complied with the ethical guidelines outlined in the World Medical Association’s Declaration of Helsinki.

A Mayo endoscopic subcore (MES) of at least 2 or a partial Mayo score (PMS) (Schroeder et al., 1987) of at least 5 for UC, and a Simple Endoscopic Score for Crohn’s Disease (SES-CD) (Daperno et al., 2004) of at least 7 (and a Rutgeerts score (Rutgeerts et al., 1990) of at least i2 for operated patients) or a Harvey-Bradshaw index (HBI) (Harvey and Bradshaw, 1980) of at least 8 for CD were required for inclusion. Adult healthy controls (HC) were also recruited from Careggi University Hospital and University of Florence staff.

The protocol was approved by the Ethical Committee of Careggi-University Hospital (CEAVC), Florence, Italy (protocol no. 19278/September 2021), and written informed consent was obtained from all participants. Demographic and clinical data were collected. Laboratory test including C-reactive protein (CRP) and faecal calprotectin (FC) were collected when required according to the standard of care. All enrolled patients underwent a colonoscopy both at baseline and at week 52. The exclusion criteria for both IBD patients and HC were other coexisting systemic diseases (e.g., diabetes, cardiovascular diseases, infectious diseases), malignancies, pregnancies, presence of oral cavity diseases such as periodontitis and gingivitis or the use of antioxidant supplements. Patients with oral manifestations of IBD (e.g., cobblestoning, orofacial granulomatosis, lip swelling with fissuring) or with dry mouth were also excluded from the study.

Treatment schedules were the following:

- IFX: 5 mg/kg intravenously at week 0, 2, 6 and then every 8 weeks.
- VDZ: 300 mg intravenously at week 0, 2, 6 and then every 8 weeks.

At each infusion, patients underwent a clinical examination to record HBI and PMS.

Drug response criteria for UC were defined as follows: a) Clinical response at week 26: PMS decreased by ≥ 3 points from baseline, or remission (PMS of 2 or less). b) Endoscopic response at week 52: MES decreased by 1 point from baseline, or remission (MES of 0–1).

Drug response criteria for CD were defined as follows: a) Clinical response at week 26: HBI decreased by ≥ 3 points from baseline, or remission (HBI of 4 or less). b) Endoscopic response at week 52: SES-CD score decreased by ≥ 50% from baseline, or remission (SES-CD of 0–2, in the absence of ulcers).

### 2.2 Saliva collection

All participants were instructed to collect at least 1 mL of whole unstimulated saliva, in the morning, before eating, and at least 30 min after tooth-brushing. Saliva was centrifuged at 5,000 *g* for 10 min, at 4 °C. The supernatant was then dived into aliquots and stored at – 20 °C.

### 2.3 Ferric reducing activity of saliva (FRAS)

The FRAS reagent solution was freshly prepared by mixing 300 mM acetate buffer, pH 3.6, TPTZ solution (10 mM 2,4,6-tripyridyl-s-triazine (TPTZ) in 40 mM HCl), and 20 mM FeCl_3_·6H_2_O in a volume ratio of 10:1:1. To perform the assay, 0.9 mL of FRAS reagent, 90 µL of distilled water, and 10 µL of saliva were mixed and the absorbance was measured at 595 nm. The antioxidant potential was calculated from a standard curve plotted using FeSO4·7H2O (Benzie and Strain, 1996).

### 2.4 Advanced oxidation protein products (AOPPs)

For AOPPs measurement, 100 µL of saliva were diluted to 1 mL with PBS, then mixed to 50 µL of 1.16 M KI and 100 µL of glacial acetic acid. The absorbance of the reaction mixture was immediately read at 340 nm. AOPPs were quantified using Chloramine-T (Sigma-Aldrich, Milan, Italy) as reference standard and quantity expressed as nmol/mg of proteins (Witko-Sarsat et al., 1996). Protein content was estimated by using the Bio-Rad DC protein assay kit (Bio-Rad, Segrate, Milan, Italy).

### 2.5 Advanced glycation end products (AGEs)

AGEs were determined according to Cournot et al. (2018). Salivary samples (100 µL) were placed in a 96-well black plate and the fluorescence intensity read at 460 nm, after excitation at 355 nm. Data were expressed as arbitrary units (AU).

### 2.6 Statistical analyses

Statistical analyses were performed using GraphPad Prism 8.02. Normality testing was performed using Shapiro-Wilk and D'Agostino-Pearson tests. Continuous variables were presented as medians and interquartile range (IQR), whereas frequencies and proportions were used for categorical variables. Comparisons among groups for continuous variables were performed using Mann–Whitney tests, Kruskal–Wallis tests or Wilcoxon matched-pairs signed rank tests, while for categorical variables Fisher’s exact test or χ^2^ test were performed, as appropriate.

The diagnostic utility of AOPPs and AGEs in reference to CRP and FC was tested using Receiver operating characteristic (ROC) curve analyses. Performance was expressed as the area under the ROC curve (AUC) with 95% CI. A cut-off value corresponding to the highest accuracy (minimal false positives and false negatives) was determined by Youden’s index calculation (sensitivity + specificity - 1) for comparative purposes (Xing et al., 2025). P values <0.05 were considered significant.

## 3 Results

### 3.1 Baseline characteristics of IBD patients and healthy controls

In total, 73 IBD patients (of whom 27 with CD and 46 with UC) and 56 healthy controls (HC) were included; no significant gender and age distribution difference was observed between HC and IBD patients nor between CD and UC. However, 23% of HC were active smokers compared to 6.8% of IBD patients (p = 0.0002) (Table 1).

**TABLE 1:** Baseline characteristics of IBD patients and healthy controls.

	HC (n = 56)	IBD (n = 73)	p-value ^a^
**Age, y**	60 (18–65)	49 (41–64.5)	0.1182
**Gender, F, n (%)**	39 (60.9%)	37 (50.7%)	0.3013
**Smoking habits, n (%)**			**<0.0001**
Non-smokers	49 (76.6%)	51 (69.9%)	
Current smokers	15 (23.4%)	5 (6.8%)	
Former smokers	0	17 (23.3%)	
**AOPPs (nmol/mg proteins)**	17.3 (8.72–31.79)	75.0 (47–200.1)	**<0.0001**
**AGEs (AU)**	38.5 (21.5–56.65)	60.0 (44.5–125.5)	**<0.0001**
**FRAS (µM)**	324.0 (164.0–501.0)	326.0 (205.0–569.3)	0.4241

Data are presented as median and IQR, or proportions (n, %). Bold font indicates statistical significance. HC, healthy controls; IBD, inflammatory bowel diseases patients; AOPPs, Advanced Oxidation Protein Products; AGEs, Advanced glycation-end products.

^a^ Fisher’s exact test or χ2 test for the categorical variables and Mann-Whitney’s test for the continuous variables.

All CD patients were treated with IFX, whereas half of the UC patients received VDZ therapy (n = 23). At baseline colonoscopy, the median SES-CD score was 6 (IQR 1.75–10) with three operated patients with Rutgeerts score i2 and 5 with i3-4, while the median MES score was 3 (IQR 2–3). The median clinical activity in CD patients was 4 (IQR 2–5) according to HBI and 4 (IQR 2–6) for UC patients according to PMS (Table 2).

**TABLE 2:** Characteristics of CD and UC patients at baseline, at week 26 and at week 52.

CD (n = 27)	Baseline	Week 26	Week 52
CRP (mg/dL)	0.5 (0.4–1.45)	0.5 (0.2–0.6)	0.5 (0.5–0.7)
FC (µg/g)	247 (83–479)	98 (19–360)	67 (50–297.3)
HBI	4 (2–5)	1 (0–3) **	0 (0–1) ****
SES-CD	6 (2–10)		2 (0–4) *
Rutgeerts score			
*i2, n*	3 (37.5%)		7 (87.5%)
*i3-4, n*	5 (62.5%)		1 (12.5%)
Clinical Response, n (%)		21 (77.8%)	
Therapy ongoing n (%)		23 (85.2%)	
Months in therapy			33 (6–39)
Endoscopic improvement, n (%)			14 (51.9%)
Endoscopic remission, n (%)			11 (40.7%)
AOPPs (nmol/mg proteins)	132.3 (70.8–222.7)	121.9 (74.7–208.1)	
AGEs (AU)	72 (52.1–107)	63.5 (36.4–72.2) **	

UC (n = 46)	Baseline	Week 26	Week 52
CRP (mg/dL)	0.7 (0.5–1.12)	0.5 (0.5–0.89)	0.5 (0.5–0.5)
FC (µg/g)	550 (366–1859)	118 (49.6–517) ***	74.5 (34.5–601) ***
PMS	4 (2–6)	0 (0–1) ****	0 (0–1) ****
MES	3 (2–3)		2 (0.3) ***
Clinical Response, n (%)		32 (69.6%)	
Therapy ongoing, n (%)		37 (80.4%)	
Months in therapy			20.5 (6–36)
Endoscopic improvement, n (%)			20 (43.5%)
Endoscopic remission, n (%)			11 (23.9%)
AOPPs (nmol/mg proteins)	70.4 (28.5–182.7)	81.4 (48.6–170.4)	
AGEs (AU)	57 (43–148)	49 (40–136)	

Data are presented as median and or proportions (n, %). CRP, C Reactive Protein; FC, faecal calprotectin; HBI, Harvey–Bradshaw index; PMS, partial Mayo score; AOPPs, Advanced Oxidation Protein Products; AGEs, Advanced glycation end products; *p < 0,05; ***p < 0.001; ****p < 00,001 vs. baseline.

### 3.2 Baseline AOPPs and AGEs and correlations with endoscopic disease activity

At baseline, despite the higher prevalence of smokers in the control group (23.4% vs. 6.8%), IBD patients had higher levels of salivary AOPPs and AGEs than HC (p < 0.0001) (Table 1), whereas their FRAS values were similar (Table 1).

Salivary levels of AOPPs, but not AGEs, significantly discriminated patients with mild endoscopic activity from those with moderate-to-severe disease with an area under the curve (AUC) of 0.72, (95% confidence interval (CI) 0.54–0.91, p < 0.05, Table 3). For AOPPs, the optimal cut-off value with the highest YI (0.49) was set at >71.95 ng/mg proteins with a sensitivity of 85.71% and a specificity of 63.16% (Table 3). For comparison, neither CRP nor FC discriminated patients with mild endoscopic activity from those with moderate-to-severe disease: the AUC of CRP was in fact 0.60 (95% CI 0.45–0.76, p = 0.21) while that of FC was 0.67 (95% CI 0.50–0.85, p = 0.08 (Table 3; Supplementary Figure S1).

**TABLE 3:** Performance of Advanced Oxidation Protein Products (AOPPs) and Advanced glycation end products (AGEs) in discriminating disease activity compared to C Reactive Protein (CRP) and faecal calprotectin (FC).

	AOPPs	AGEs	CRP	FC
Mean AUC (95% CI)	0.72 (0.54–0.91) **p = 0.03**	0.52 (0.29–0.75) p = 0.90	0.60 (0.45–0.76) p = 0.21	0.67 (0.50–0.85) p = 0.08
Cutoff value	71.95	61.0	0.65	447.0
Sensitivity %	85.71	53.33	56.00	68.42
Specificity %	63.16	63.64	68.00	55.56
YI	0.49	0.17	0.24	0.24

AUC, area under receiver operating characteristic (ROC) curve; CI, confidence interval; YI, Youden’s index calculated as: sensitivity + specificity −1. Bold font indicates statistical significance.

### 3.3 Association between AOPPs, AGEs and response to treatment

At week 26 of treatment, 77.8% of CD patients and 69.6% of UC patients showed clinical response, with HBI (p < 0.05) and PMS (p < 0.0001) values significantly decreased compared to baseline (Table 2). The median levels of salivary AOPPs were unchanged compared to baseline, while those of AGEs were slightly reduced (p < 0.05). No significant difference in the median levels of AOPPs or AGEs was found between clinical responders and non-responders (data not shown). At week 52, improved endoscopic disease activity was observed in 51.9% of CD patients, and 40.7% achieved mucosal healing. Among UC patients, 43.5% had improved endoscopic disease activity, while only 23.9% were in endoscopic remission (Table 2). Neither baseline nor 6-months follow-up levels of AOPPs and AGEs nor their variations (Δ) were predictive of endoscopic response or remission at week 52. For UC patients, we also performed a subgroup analysis to assess the effects of IFX and VDZ on clinical and endoscopic responses, as well as on changes in salivary oxidative stress, and found no significant differences (data not shown).

## 4 Discussion

ROS are essential for immune responses, but their overproduction triggers and perpetrates proinflammatory pathways, particularly trough redox sensitive transcription factors nuclear factor (erythroid-derived 2)-like 2 (Nrf2) and nuclear factor-κB (NF-κB) (Muro et al., 2024). Inflammation further amplifies oxidative stress by increasing ROS production and myeloperoxidase release, causing oxidative damage to lipids, nucleic acids and proteins and contributing to intestinal tissue injury (Muro et al., 2024). Previous studies, including our own, have shown elevated plasma levels of AOPPs and AGEs in IBD patients (Luceri et al., 2019; Krzystek-Korpacka et al., 2008). However, evidence is still lacking on whether salivary levels of these markers reflect clinical or endoscopic disease activity and have potential as predictors of therapeutic response.

In this study, we demonstrated that both salivary AOPPs and AGEs levels are significantly higher in IBD patients compared to healthy controls despite the higher percentage of smokers in the latter group. This agrees with the results of Janšáková et al. (2021) who found higher salivary AOPPs and AGEs levels in CD patients compared to controls. Salivary AOPPs levels also showed satisfactory performance in discriminating severe from mild-moderate disease activity. ROC curve analysis demonstrated that baseline salivary AOPPs levels performed better than FC, the gold standard marker for disease activity, indicating the overall accuracy of AOPPs in reflecting disease status.

Besides being a product of oxidative damage and inflammation, AOPPs appear to have a pathogenic role as ligands for RAGE and CD36 receptors that mediate AOPP- induced intestinal epithelial cell cycle arrest (Shi et al., 2019). AOPPs have been found to trigger inflammation and intestinal epithelial cell death through a redox-dependent pathway, mediated by c-jun N-terminal kinase (JNK) and polymerase-1 (ADP-ribose) (PARP-1) (Xie et al., 2014). Chronic administration of AOPPs induced epithelial-mesenchymal transition in intestinal cells, negatively affecting intestinal integrity (Xu et al., 2017). More recently, it was shown that AOPPs accumulate in the inflammatory cells of the intestinal crypts of CD patients, and that AOPPs treatment caused defects in Paneth cells by inducing endoplasmic reticulum stress and mitochondrial dysfunction (Shi et al., 2023).

Given their pathogenic role, we also explored whether AOPPs might serve as useful redox biomarkers to monitor disease activity and therapy response; to this aim, the levels of AOPPs were serially assessed at baseline and after 26 weeks of IFX or VDZ therapy, when clinical response was evaluated. This is the first prospective study to demonstrate that, even when clinical response to IFX and VDZ is achieved, elevated levels of salivary AOPPs persist. This finding strengthens existing data of reduced antioxidants in patients in clinical remission (Bourgonje et al., 2019) further suggesting that increased oxidative stress characterises subclinical disease activity and may trigger subsequent disease recurrence.

Since the failure rate of IFX or VDZ is approximately 50%–60% (Raine and Danese, 2022), identifying biomarkers that can predict endoscopic remission is a high-priority area of research in personalized treatment strategies. In this study, a high percentage of patients treated with IFX or VDZ did not achieve endoscopic improvement at week 52 and even a higher percentage of patients did not reach endoscopic remission.

Notably, salivary AOPPs levels, both at baseline and after 6 months of treatment, failed to demonstrate predictive value for therapeutic response to IFX or VDZ, thereby restraining their clinical applicability as non-invasive biomarkers. Nevertheless, our data indicate that AOPPs accumulate in patients with IBD, reflecting a sustained oxidative stress burden that persists despite biologic therapy with IFX or VDZ, underscoring a pathophysiological process not adequately targeted by these agents.

Recent studies highlight the role of oral microbiota in IBD, giving rise to the concept of the “gum–gut axis” to explain the reciprocal relationship between the oral cavity and the gut and its impact on disease onset and progression (Wang et al., 2024; Bird and Gulati, 2021). IBD patients often show oral dysbiosis and reduced microbial diversity (Abdelbary et al., 2022; Qi et al., 2022; Elzayat et al., 2023). The oral cavity may serve as a reservoir for microbes that translocate to the gut, contributing to intestinal dysbiosis (Abdelbary et al., 2022). Microbiota regulates redox balance by multiple mechanisms (Kunst et al., 2023): a clear example is the ability of microbial metabolites such as butyrate to inhibit the NADPH oxidase DUOX2 activity limiting barrier dysfunction and inflammation (Hazime et al., 2025). Together with metabolic factors such as fumarate that mitigate oxidative stress (Xiao et al., 2025), these mechanisms suggest that dysbiosis and metabolic imbalance may jointly sustain oxidative stress in IBD. These processes could explain the persistence of elevated AOPPs in our patients despite clinical response, suggesting oxidative stress as a potential therapeutic target through modulation of the oral–gut axis and/or antioxidant interventions.

Given the paucity of randomized controlled trials investigating antioxidants as add-on therapy, it remains uncertain whether directly targeting oxidative stress improves response rates. Nonetheless, some studies suggest that antioxidants may complement standard therapy: N-acetyl-L-cysteine or curcumin co-administered with 5-ASA improved clinical response in UC patients (Lang et al., 2015; Guijarro et al., 2008), vitamin B2 alongside thiopurines and/or anti-TNFα reduced oxidative stress and clinical symptoms in CD patients (von Martels et al., 2020), and antioxidant anthocyanins enhanced IFX-mediated disease remission in terms of circulating inflammatory markers in a small pilot study (Liso et al., 2022).

## 5 Conclusion

In conclusion, we found that salivary AOPPs levels are elevated in patients with IBD and correlate with endoscopic disease severity at baseline. Moreover, AOPPs remain persistently high after 26 weeks of treatment with IFX or VDZ, irrespective of the clinical or endoscopic response at 52 weeks, suggesting that oxidative stress may not be adequately controlled by these therapeutic agents. The limitations of single-center recruitment and the relatively modest cohort size should be acknowledged, as they may restrict the generalizability of these findings and warrant confirmation in larger, multicenter studies.

However, our findings highlight the need for future studies to clarify the clinical relevance of persistent AOPPs and their main drivers, including oral–gut dysbiosis, metabolic regulators, and the underlying molecular mechanisms. Such investigations are essential before considering potential therapeutic applications of antioxidant-based adjunctive therapies or interventions targeting AOPP-mediated damage to enhance remission rates of advanced treatment in IBD.

## Data Availability

The original contributions presented in the study are included in the article/Supplementary Material, further inquiries can be directed to the corresponding author.
