# Supplementary figures and images for: Salivary oxidative stress persists in inflammatory bowel disease regardless of biological treatment response
Source: Front Pharmacol. 2025 Nov 7;16:1699252. doi: 10.3389/fphar.2025.1699252 (PMC12634346; doi:10.3389/fphar.2025.1699252)

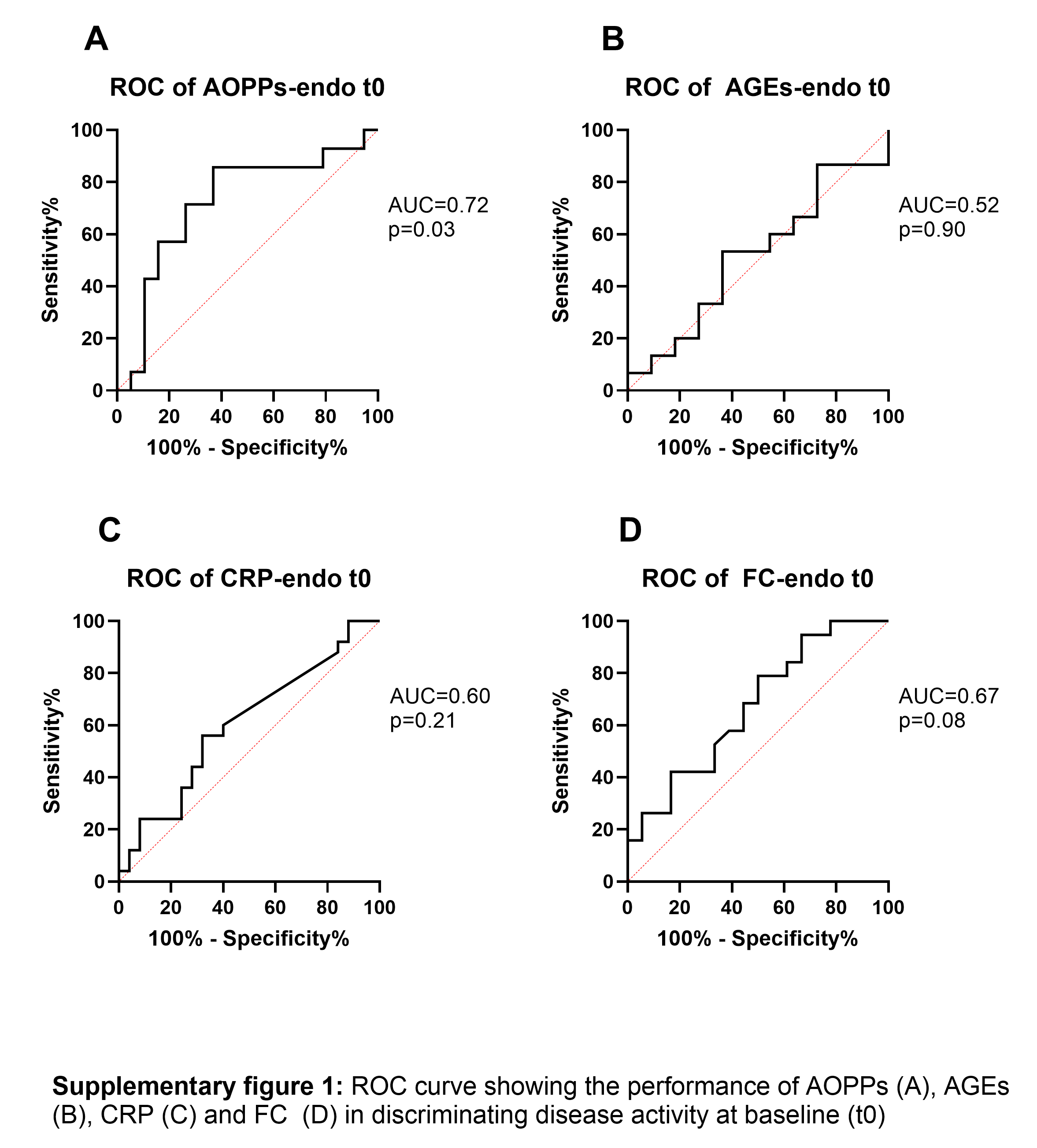

Supplement: Supplementary file 1 [file Image1.tif]
